# From Across the Seas

**Published:** 1917-04-14

**Authors:** 


### FROM ACROSS THE SEAS.

### The Position of Margarine in the Food World.

The March number of *The Modern Hospital*, which has removed its offices to Conway Building, 111 West Washington Street, Chicago, contains a useful article by Mr. J. P. Street on the composition of margarine, or as it is called in the United States, oleo-margarine, the process of its manufacture and its position in the food world. This article of diet, which during the war has been so widely accepted as a butter substitute, owes its origin to the necessities produced by another war, the Franco-Prussian War of 1870, when a French chemist, Megé-Mouries, first produced an artificial butter from beef fat. Numerous patents have been granted since then, but the essential features of the process have shown but little change since 1870. The average composition of a high-grade butter and a high-grade margarine are stated to be as follows:—

| | Butter, per cent. | Margarine, per cent. |
|---|---|---|
| Water ... ... ... | 11.0 | 8.6 |
| Protein ... ... ... | 1.0 | 0.9 |
| Fat ... ... ... | 85.0 | 88.8 |
| Ash (chiefly salt) ... | 3.0 | 1.7 |

The writer of the article claims that when margarine is made only from high-grade, wholesome raw material, and is manufactured in a clean and sanitary manner, it contains fully as much nutriment as butter, and is almost as digestible.

### News from New Zealand.

A conference of delegates from Hospital and Charitable Aid Boards throughout the Dominion of New Zealand met early in January to consider, amongst other matters, the question of formulating a co-operative scheme for purchasing hospital supplies. Mr. H. Baldwin, chairman of the Wellington Board, was voted to the chair, and a representative attendance included the Hon. G. W. Russell, Minister for Public Health, and Dr. Valintine, Inspector-General of Hospitals. The conference adopted a report from a committee, which had conferred with the Minister, which recommended that boards should prepare schedules of supplies obtained in the past year under the headings of drugs and surgical requisites, furniture and hospital equipment, and other recurring items. The committee did not consider it possible at present to apply for a scheme of pensions. In respect of the purchase of supplies it was agreed that a committee be set up to deal with purchases confined to lines most largely used in all hospitals. For goods obtainable within the Dominion tenders are to be invited by this committee, and by the High Commissioner for those imported. In respect of imported goods it was suggested by the Minister of Public Health that an agreement might be made for remission of duty. It was decided to endeavour to obtain for hospitals the reversion of motor-ambulance wagons now used for war purposes, and that the Government be asked to amend the law in such a way as to provide that a more equitable rate of subsidy be paid throughout the Dominion on all contributions paid by contributory local authorities on account of expenditure other than capital expenditure.

### Hospital Workers' Trade Union.

The *Royal Prince Alfred Hospital Gazette*, of Sydney, publishes in full an agreement entered into between the hospital and the Hospital and Asylum Employees' Union binding the parties concerned for three years. As in this country, at any rate as regards voluntary hospitals, institutional workers are not associated in any general union or society, it may interest our readers to learn what combined requests have obtained in Australia in respect of some of the terms of service. The hours of working (exclusive of meal times) shall not exceed forty-eight per week when paid at ordinary rates, overtime (as specified) shall be paid at time and a-half, this arrangement including work in the venereal clinic at night, in addition to ordinary duties. Nine public holidays are enumerated (including King's Birthday, Prince of Wales's Birthday, and Eight-Hour Day), which are to be allowed to workers alternately on full pay. Wardmaids may be called to work up to 2 p.m. on public holidays. After a year's service employees are to be allowed twelve days' leave of absence on full pay. Members and non-members of the union employed together shall work in harmony. Exemptions from the agreement include members of the medical and nursing staffs and the secretary or his deputy.

